# Neurodevelopment and Gross Motor Function Outcomes in Children at Risk or Diagnosed with Cerebral Palsy using Snoezelen Multisensory Room

**DOI:** 10.21315/mjms-04-2025-289

**Published:** 2025-12-31

**Authors:** Ameerah Ali, Surini Yusoff, Wan Mohd Aiman Wan Ab Rahman, Hans Van Rostenberghe, Najib Majdi Yaacob, Fatimah Az-Zahraa’ Dinsuhaimi

**Affiliations:** 1 Department of Paediatrics, School of Medical Sciences, Universiti Sains Malaysia, Health Campus, Kelantan, Malaysia; 2 Rehabilitation Medicine Unit, Hospital Pakar Universiti Sains Malaysia, Kelantan, Malaysia; 3 Unit of Biostatistics and Research Methodology, School of Medical Sciences, Universiti Sains Malaysia, Health Campus, Kelantan, Malaysia; 4 International Medical School, Management and Science University, Shah Alam, Malaysia; 5 Rehabilitation Medicine Unit, School of Medical Sciences, Universiti Sains Malaysia, Health Campus, Kelantan, Malaysia

**Keywords:** cerebral palsy, multi-sensory room, neurodevelopmental, gross motor function, rehabilitation

## Abstract

**Background:**

The Snoezelen multisensory room is an internationally recognised intervention designed to provide sensory stimulation that promotes developmental progress in children. This study aimed to evaluate neurodevelopmental and gross motor outcomes in children at risk of, or diagnosed with, cerebral palsy and who received neurodevelopmental therapy in the Snoezelen multisensory room at Hospital Pakar Universiti Sains Malaysia, Kelantan.

**Methods:**

Twenty-six patients aged three to 24 months, either at risk of or diagnosed with cerebral palsy, were recruited. However, only 20 patients completed the study. All participants underwent neurodevelopmental therapy in the Snoezelen multisensory room for 12 weeks. Demographic and clinical characteristics were recorded. Neurodevelopment was assessed using the Hammersmith Infant Neurological Examination (HINE), and gross motor function was evaluated using the Gross Motor Function Measure (GMFM-88) scores. Assessments were performed before therapy and after 12 weeks. Pre- and post-therapy scores were compared using paired *t*-tests.

**Results:**

HINE scores increased significantly from pre-therapy (mean = 60.70, standard deviation [SD] = 16.19) to post-therapy (mean = 66.75, SD = 18.18; *t* = −3.211, *P* = 0.005). GMFM-88 scores also showed significant improvement from pre-therapy (mean = 49.00, SD = 34.34) to post-therapy (mean = 101.95, SD = 55.70; *t* = −6.116, *P* < 0.001).

**Conclusion:**

Neurodevelopmental therapy delivered in a Snoezelen multisensory room was associated with significant improvements in neurodevelopmental and gross motor function after 12 weeks among children at risk of cerebral palsy.

## Introduction

Cerebral palsy (CP) is a group of permanent yet dynamic disorders affecting movement, posture, and motor function, resulting from non-progressive interference, lesions, or abnormalities in the developing brain (1). The diagnosis is primarily based on motor and postural dysfunctions that appear in early childhood and persist throughout life. Motor impairments, the hallmark of CP, are often accompanied by additional challenges such as sensory, perceptual, cognitive, communication, and behavioural disorders, as well as epilepsy and secondary musculoskeletal complications (1). The prevalence of CP in developed countries is estimated at 1.5 to 2.0 per 1,000 live births. In Malaysia, although data on CP prevalence are limited, it is believed to be slightly higher. In 2012, the Ministry of Health recorded 215 children with CP in the National Disability Registry (2).

The Snoezelen multisensory room is now a well-recognised therapeutic approach among healthcare providers. Developed in the late 1970s, Snoezelen was initially designed for individuals with severe to profound intellectual and multiple disabilities (3). The concept is based on the understanding that such individuals perceive and interact with their environment primarily through sensory experiences. Due to limited independent mobility and heightened sensory sensitivity, they may struggle to engage with or tolerate the demands of typical environments (3).

Studies have demonstrated various potential benefits of Snoezelen therapy in individuals with disabilities. In occupational therapy interventions, the Snoezelen multisensory room has gained recognition as a therapeutic environment that stimulates the senses and promotes relaxation, exploration, and engagement in individuals with developmental, cognitive, or sensory impairments (4). The intervention is conducted in a specially designed sensory room equipped with elements such as fibre-optic lights, bubble tubes, tactile panels, and soft music (5). Although its use and effectiveness have been explored in a wide range of populations globally, including those with developmental disabilities, evidence from Malaysia remains limited.

Caring for a child with CP is a long-term process aimed at ensuring the best possible quality of life for both the child and their family. A key aspect of CP management is holistic functional improvement, with systematic and intensive motor rehabilitation playing a central role. This rehabilitation is based on the principle of neuroplasticity — the nervous system’s ability to undergo lasting structural and functional changes in response to internal and external stimuli. The greatest potential for modification occurs during the early stages of central nervous system development when the brain exhibits high plasticity, allowing for better compensation of deficits. Therefore, rehabilitation and early intervention programmes for children at risk of CP should begin as early as possible (1, 6). However, most studies evaluating the effectiveness of Snoezelen therapy have focused on children with autism spectrum disorder or premature infants, while research involving children with CP remains scarce and largely retrospective (5, 7, 8).

For instance, a survey conducted at Cardiff University, United Kingdom, demonstrated improved sensory regulation among children with autism, enhancing their ability to maintain attention during learning activities (9). Similarly, a study involving autistic adolescents and adults in Serbia reported a reduction in the severity of repetitive and stereotyped behaviours (10), and research from Iran found that multisensory stimulation positively affected neuromuscular development in premature infants (11).

In Malaysia, Snoezelen multisensory rooms have been introduced through the efforts of governmental and non-governmental organisations. Within the state of Kelantan, the authors are aware of three such facilities; however, many remain underused. This underutilisation may be due to a lack of awareness among healthcare professionals and a lack of local evidence.

The objective of this study is to determine neurodevelopmental and gross motor function outcomes, measured using the Hammersmith Infant Neurological Examination (HINE) and Gross Motor Function Measure (GMFM-88), respectively, among children at risk of or diagnosed with CP before and after receiving therapy in a Snoezelen multisensory room. This study aims to provide clinicians with local evidence to support the effective use of available facilities.

## Methods

### Study Design

This was a prospective cohort study involving children at risk of, or diagnosed with, CP and who were under follow-up at the Paediatric Clinic, Hospital Pakar Universiti Sains Malaysia (HPUSM).

### Subjects

**Figure 1:**
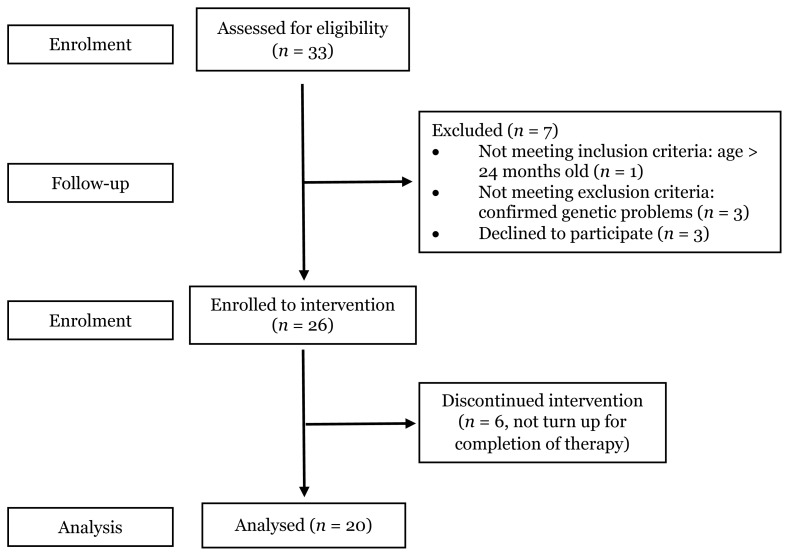
Study flow chart

The study population comprised children aged three to 24 months. All eligible patients were recruited using universal sampling, and informed consent was obtained from their parents or caregivers (Figure 1).

The inclusion criteria were as follows:

- Age between three and 24 months (for premature infants, corrected age was calculated as chronological age minus the number of weeks born before 40 weeks’ gestation).
- Under follow-up at the Paediatric Clinic, HPUSM.
- A confirmed diagnosis of CP, as established by a paediatrician, or an increased risk of CP, defined by (12):
  - Gross motor delay, hand function asymmetry, or tiptoeing.
  - The presence of antenatal, perinatal, or postnatal risk factors.

Exclusion criteria were as follows:

- Confirmed genetic disorders.
- A history of seizures within the past three months, as sensory lights in the room could trigger seizures (13).
- Moderate to severe visual or hearing impairments, which would hinder optimal sensory engagement during therapy.
- Dyskinetic CP, as these children may find it difficult to control movements and comply with the therapy protocol (14).

### Sample Size

The sample size was calculated based on a paired *t*-test design, with an expected mean difference of three units, a standard deviation (SD) of 6.5, a significance level (α) of 0.05 (two-tailed), and a desired power of 80% (*β* = 0.20). Based on these parameters, the minimum required sample size was 36 pairs. To allow for an estimated 10% dropout rate, the final target sample size was increased to 40 participants. However, only 20 participants were included due to logistical challenges and time constraints that prevented some from continuing the study. Although this reduced the statistical power of the study to approximately 0.7, the data still provide valuable preliminary insights into the neurodevelopmental and gross motor outcomes of children at risk of, or diagnosed with, CP.

### Research Tools

Demographic and clinical characteristics were collected using a standard pro forma, along with the HINE and GMFM-88 scores. The HINE is a standardised neurological examination for infants aged two to 24 months and is predictive of CP. It evaluates cranial nerve function, posture, movements, reflexes, and muscle tone, with total scores ranging from 0 to 78 (15, 16). The GMFM-88 is a standardised observational tool used to evaluate and monitor changes in gross motor function in children with CP aged five months to 16 years. It includes five dimensions: “lying and rolling,” “sitting,” “crawling and kneeling,” “standing,” and “walking, running, and jumping,” with total scores ranging from 0 to 264. GMFM-88 is widely used in research to assess treatment efficacy, compare therapeutic approaches, and explore the natural history of motor development in children with CP (17).

### Procedures

The study was conducted in the Snoezelen multisensory room at HPUSM between June 2024 and February 2025. HINE and GMFM-88 scores were assessed before therapy and 12 weeks after therapy initiation by the principal investigator or one of two dedicated occupational therapists. All assessors underwent training by an expert in the field, and interrater reliability was confirmed at the start of the study (identical scores were obtained for a representative patient).

Participants underwent several sessions of neurodevelopmental treatment (NDT) in the Snoezelen multisensory room. The NDT approach is individualised and tailored to each child’s specific problems (18). The occupational therapist facilitated movement using key points of control, such as the head, shoulders, and pelvis, to guide coordinated movement of the entire body (18). The frequency of therapy sessions varied for each patient, depending on their scheduled appointments with the occupational therapist and adherence to the sessions. Each session lasted approximately one hour, and reassessment was conducted after 12 weeks of therapy.

### Statistical Analysis

Descriptive statistics were used to summarise sociodemographic and clinical characteristics. Categorical variables were presented as frequencies and percentages, whereas continuous variables were expressed as means ± SD or medians with interquartile ranges (IQR), depending on data normality. Normality of continuous variables was assessed using the Shapiro–Wilk test and by visual inspection of histograms and Q–Q plots. Variables with *P*-value < 0.05 on the Shapiro–Wilk test were considered non-normally distributed. Based on data distribution, appropriate parametric or non-parametric tests were applied. The paired *t*-test (for normally distributed data) or the Wilcoxon signed-rank test (for non-normal data) was used to compare pre- and post-intervention scores. Correlations between pre-and post-intervention scores were examined using Pearson’s correlation coefficient (for normal data) or Spearman’s rank correlation coefficient (for non-normal data). All analyses were conducted using the Statistical Package for the Social Sciences (SPSS) version 28. A *P*-value of less than 0.05 was considered statistically significant. Analyses were conducted on complete cases only; participants with missing follow-up data were excluded.

## Results

During the study period from June 2024 to February 2025, 33 patients under paediatric follow-up at HPUSM were screened. Four patients did not meet the inclusion criteria, and three parents declined consent. A total of 26 participants were enrolled, but six subsequently dropped out. Thus, 20 patients completed the study and underwent both pre-and post-intervention assessments.

The sociodemographic and clinical characteristics of the participants are summarised in Table 1. The median age was 12 months (range: three to 23 months), and the male-to-female ratio was 1:1. All participants were Malay. Only two patients (10.0%) had a confirmed diagnosis of cerebral palsy, while the remaining 90.0% were considered at risk of developing CP due to gross motor delay, global developmental delay, and/or perinatal risk factors. As shown in Table 1, prematurity was the most common contributing factor, accounting for 50.0% of cases. Half of the participants’ caregivers (50.0%) had secondary-level education, and 45.0% had tertiary education. Most participants (85.0%) were from low-income (B40) households, with the remainder from middle-income (M40) families. All participants received neurodevelopmental treatment tailored to their clinical needs.

The Shapiro–Wilk test indicated that the data were normally distributed. Therefore, parametric tests were applied: paired *t*-tests for pre- and post-intervention comparisons and Pearson’s correlation for correlation analysis. The pre- and post-intervention scores (Table 2) showed statistically significant improvements for both HINE and GMFM-88.

Therapy frequency varied according to appointment schedules and participant adherence. Each session lasted one hour. Only two participants (10.0%) attended five sessions, while the majority attended between two and four sessions. Table 3 shows the correlation between therapy frequency and improvement in HINE and GMFM-88 scores; these correlations were not statistically significant.

## Discussion

This study is the first in Southeast Asia to evaluate the effects of neurodevelopmental therapy in a Snoezelen multisensory room for children at increased risk of, or diagnosed with, CP. The results provide preliminary evidence that such therapy may improve neurodevelopmental and gross motor function after 12 weeks. Improvements in neuromuscular development following multisensory stimulation have been demonstrated previously, including in a randomised clinical trial involving preterm infants in Iran (11). Given that prematurity was the most common risk factor in our study population, our findings further support the potential benefits of multisensory stimulation beyond the neonatal period. Similarly, our results resonate well with a Korean study that utilised a multisensory approach to improve gross motor function in children with CP (7). Participants in that study also demonstrated increased participation in the activity. This suggests that enhanced participation may contribute to improved motor outcomes.

The findings of the present study differ from those of a retrospective study conducted in Mexico (19). The variation in results is likely due to methodological differences and disparities in patient characteristics. In the Mexican study, overall improvement was not significant, possibly because nearly half of the patients had established CP. However, subgroup analysis of patients merely at risk of CP revealed significant motor improvements, which is consistent with our findings. This suggests that children at risk of CP may benefit more from multisensory stimulation than those with a confirmed diagnosis. The improvements are also plausible given the heightened neuroplasticity during the first three years of life, when the brain is most responsive to environmental enrichment and sensory input.

This preliminary study has several limitations. First, the sample size was small (*n* = 20), which reduced statistical power compared to the target sample size of 40 and may limit generalisability and reliability of the findings. Second, the study lacked a control group, which restricts the ability to attribute improvements solely to the intervention. Third, the dropout rate exceeded 20%, potentially influenced by social problems and logistical challenges of patients who need to visit the hospital. Previous work from the same centre has shown non-attendance rates exceeding 30% (20). Lastly, the study duration was limited to 12 weeks; therefore, longer-term effects of Snoezelen multisensory therapy could not be assessed. Future longitudinal studies with larger sample sizes and a controlled design are recommended.

## Conclusion

In conclusion, this study suggests that neurodevelopmental therapy delivered in a multisensory Snoezelen room may have positive effects on neurodevelopment and gross motor function, particularly for children at risk of CP.

## Figures and Tables

**Table 1:** Sociodemographic and clinical characteristics of participants

Variables	Results	

	*n* = 26	*n* = 20
Age (months), median (IQR), (min, max)	12 (3, 23)	12 (3, 23)

Gender, no. (%)		
Male	12 (46.2)	10 (50.0)
Female	14 (53.8)	10 (50.0)

Race, no. (%)		
Malay	26 (100.0)	22 (100.0)
Others	0	0

Diagnosis, no. (%)		
Cerebral palsy	2 (7.7)	2 (10.0)
Risk of cerebral palsy	24 (92.3)	18 (90.0)

Risk factors, no. (%)		
Intrauterine growth restriction	1 (3.8)	1 (5.0)
Intrauterine infection	1 (3.8)	1 (5.0)
Prematurity	11 (42.3)	10 (50.0)
Hypoxic-ischaemic encephalopathy	2 (7.7)	2 (10.0)
Persistent pulmonary hypertension of the newborn	1 (3.8)	-
Congenital heart disease	2 (7.7)	2 (10.0)
Congenital hydrocephalus	2 (7.7)	2 (10.0)
Diabetic embryopathy	2 (7.7)	1 (5.0)
Nil	4 (15.4)	1 (5.0)

Caregiver education level, no (%)		
Primary	1 (3.8)	1 (5.0)
Secondary	14 (53.8)	10 (50.0)
Tertiary	11 (42.3)	9 (45.0)

Household income, no (%)		
B40 (less than RM 4,850)	22 (84.6)	17 (85.0)
M40 (RM 4,851 to RM 10,970)	4 (15.4)	3 (15.0)
T20 (above RM 10,971)	-	-

Median (IQR, min/max) for numerical variables; Frequency and percentages for categorical variables

**Table 2:** Comparison of HINE and GMFM-88 scores between pre- and post-intervention

Variable	Mean (pre) ± SD	Mean (post) ± SD	Mean difference	*t*-value	df	*P*-value (sig. 2-tailed)
HINE	60.70 ± 16.19	66.75 ± 18.18	−6.05 ± 8.426	−3.211	19	0.005
GMFM-88	49.00 ± 34.34	101.95 ± 55.70	−52.95 ± 38.72	−6.116	19	< 0.001

SD = standard deviation); df = degrees of freedom

**Table 3:** Correlation between therapy frequency and improvement in HINE and GMFM-88 scores

Variables	Frequency of therapy in 12 weeks	

	Pearson’s correlation (*r*)	*P*-value
HINE difference	0.298	0.201
GMFM-88 difference	0.197	0.404
